# Multifaceted Role of Pneumolysin in the Pathogenesis of Myocardial Injury in Community-Acquired Pneumonia

**DOI:** 10.3390/ijms19041147

**Published:** 2018-04-11

**Authors:** Ronald Anderson, Jan G. Nel, Charles Feldman

**Affiliations:** 1 Department of Immunology and Institute for Cellular and Molecular Medicine, Faculty of Health Sciences, University of Pretoria, Pretoria 0001, South Africa; 2 Department of Haematology, Faculty of Health Sciences, University of Pretoria and Tshwane Academic Division of the National Health Laboratory Service, Pretoria 0001, South Africa; jan.nel@up.ac.za; 3 Division of Pulmonology, Department of Internal Medicine, Charlotte Maxeke Johannesburg Academic Hospital and Faculty of Health Sciences, University of the Witwatersrand, Johannesburg 0002, South Africa; charles.feldman@wits.ac.za

**Keywords:** cardiovascular events, cholesterol-dependent cytolysins, community-acquired pneumonia, liposomes, macrolide antibiotics, neutrophils, neutrophil extracellular traps, platelets, pneumolysin antagonists, vaccines

## Abstract

Pneumolysin (PLY), a member of the family of Gram-positive bacterial, cholesterol-dependent, β-barrel pore-forming cytolysins, is the major protein virulence factor of the dangerous respiratory pathogen, *Streptococcus pneumoniae* (pneumococcus). PLY plays a major role in the pathogenesis of community-acquired pneumonia (CAP), promoting colonization and invasion of the upper and lower respiratory tracts respectively, as well as extra-pulmonary dissemination of the pneumococcus. Notwithstanding its role in causing acute lung injury in severe CAP, PLY has also been implicated in the development of potentially fatal acute and delayed-onset cardiovascular events, which are now recognized as being fairly common complications of this condition. This review is focused firstly on updating mechanisms involved in the immunopathogenesis of PLY-mediated myocardial damage, specifically the direct cardiotoxic and immunosuppressive activities, as well as the indirect pro-inflammatory/pro-thrombotic activities of the toxin. Secondly, on PLY-targeted therapeutic strategies including, among others, macrolide antibiotics, natural product antagonists, cholesterol-containing liposomes, and fully humanized monoclonal antibodies, as well as on vaccine-based preventive strategies. These sections are preceded by overviews of CAP in general, the role of the pneumococcus as the causative pathogen, the occurrence and types of CAP-associated cardiac complication, and the structure and biological activities of PLY.

## 1. Introduction

The cholesterol-binding, pore-forming toxin, pneumolysin (PLY), is recognized as being the major protein virulence factor of *Streptococcus pneumoniae* (the pneumococcus), the most common bacterial causative agent of community-acquired pneumonia (CAP) [1]. PLY not only promotes colonization of the nasopharynx by, and host-to-host transmission of, the pneumococcus [1,2,3], but also invasion of the lower respiratory tract, resulting in development of pneumonia. In patients with severe disease, this may result in acute lung injury (ALI), respiratory failure and extrapulmonary dissemination of the bacterial pathogen [1,2,4,5]. Hematogenous spread of the pneumococcus, in turn, poses the risk of distal organ invasion and development of multisystem disease. This may occur, albeit uncommonly, in the case of meningeal invasion with resultant development of meningitis [6,7], an event which is dependent on PLY-mediated injury to cerebral microvascular endothelium and neurones [2]. In this setting, PLY acts in concert with the pneumococcal adhesins, choline-binding protein A (CbpA) and phosphorylcholine, which interact with the polymeric immunoglobulin receptor and the platelet-activating factor (PAF) receptor respectively on host endothelial cells [2,8,9].

Acute and delayed-onset major cardiovascular events (CVEs), on the other hand, are now recognized as being relatively frequent extra-pulmonary complications of CAP, associated with considerable morbidity and mortality [10,11,12,13,14], with patients with pneumococcal pneumonia in particular described as being “at considerable risk for a concurrent acute cardiac event” [10]. In this context, PLY, acting via direct cytotoxic and possibly indirect pro-inflammatory/pro-thrombotic mechanisms, has been identified as a key mediator of myocardial injury [15,16], underscoring the validity of PLY as a target of adjunctive therapeutic strategies in severe pneumococcal CAP [17,18].

In recognition of the strength of recent, albeit predominantly experimental, scientific evidence, implicating PLY in the etiology of myocardial injury in pneumococcal disease, the current review represents an updated overview of this important topic. It is focused primarily on immunopathogenesis [19,20,21], as well as on PLY-targeted therapies, some of which are also broadly operative against bacterial pore-forming toxins [18,20]. These sections of the review are preceded by overviews of the epidemiology of CAP and associated CV complications, followed by a brief description of the structure and biological activities of PLY.

## 2. Community-Acquired Pneumonia (CAP) 

CAP is associated with considerable morbidity and mortality and remains the leading cause of death from an infectious disease globally, causing an estimated 3.2 million deaths annually, exceeding that of tuberculosis, human immunodeficiency virus (HIV) infection and malaria [22]. The spectrum of severity of the infection does, however, vary widely, from cases that are mild enough to be treated in the community, with a mortality of less than 1%, to severe infection, presenting as a medical emergency, with a mortality of greater than 40% [23]. The major complications of CAP that impact on prognosis are the occurrence of acute respiratory insufficiency and extra-pulmonary organ dysfunction due either to sepsis or to underlying co-morbidity [23]. 

### 2.1. Incidence of CAP

Geographic variations in the incidence of CAP have been highlighted in a recent review [24]. In Germany, an incidence of 9.7 cases per 1000 person years (translating into 660,000 patients per year) was reported, with male sex, age and co-morbidity identified as important risk factors, as well as for mortality in the case of the latter two [23,25]. In the United States, the annual incidence of CAP requiring hospitalization among adults was noted to be 24.8 cases per 10,000, rising with increasing age [26]. A more recent study documented an annual age-adjusted incidence of hospitalized patients with CAP of 649 per 100,000 adults, translating into more than 1.5 million deaths annually [27]. In one area of the United Kingdom (UK) an increase in hospitalizations for CAP since 1998 has been recorded, increasing more rapidly from 2008 onwards [28], appearing to reflect a poor understanding of the true burden of CAP, highlighting the need for accurate diagnosis and early treatment [24]. 

### 2.2. Role of Streptococcus Pneumoniae in CAP 

Although *Streptococcus pneumoniae* (pneumococcus) is the most common cause of CAP in the UK and mainland Europe [23,24,28], there has been some debate around, and further investigation into, the exact role that the pneumococcus plays in the etiology of CAP. One recent review concluded that there was a decreasing prevalence of pneumococcal infections in CAP, but that this pathogen remained the most common bacterial cause, particularly in critically ill cases, while there appeared to be an increasing role for respiratory viruses [29]. Additional studies from various geographic regions have concluded that despite the indirect protective effects of the uptake of pneumococcal conjugate vaccination in national childhood immunization programs (herd protection), the burden of pneumococcal disease remains high in adults [30,31,32,33,34]. Furthermore, it has been clearly shown that estimating the frequency of pneumococcal pneumonia from the incidence of invasive pneumococcal disease (IPD) or bacteremic pneumococcal pneumonia is a complete underestimation of the frequency of non-bacteremic pneumococcal CAP, which accounts for a much greater burden of disease and hospitalization [32,35]. It has been suggested that for every case of bacteremic pneumococcal pneumonia there are three additional cases of non-bacteremic disease [35].

Other than immunoglobulin deficiency syndromes, HIV infection and immunosenescence as major risk factors, adults with chronic conditions and who smoke, as well as those who have asthma, chronic obstructive pulmonary disease (COPD), diabetes mellitus and chronic heart disease are at increased risk of pneumococcal infection and have a higher rate of complications and mortality [36,37,38]. Clearly, these are individuals who should be targeted for pneumococcal vaccination.

Adult patients who survive an episode of IPD, as well as survivors of both bacteremic and non-bacteremic pneumococcal pneumonia, have a reduced long-term survival [39,40]. In the former study, which followed-up those who survived for three decades, most died prematurely, with a mean of 9.936 years of potential life lost [39]. The latter study, which investigated cases which survived for 30 days after presentation with non-pneumonia IPD, and both bacteremic and non-bacteremic pneumococcal pneumonia, noted that 40% died within the following 5 years, with no significant differences between the groups [40]. In both studies, increased mortality was largely related to pre-existing medical conditions. 

## 3. Cardiac Complications of CAP

It is now well recognized that cardiovascular events (CVEs) represent one of the most important and frequent complications of CAP, occurring in up to 30% of cases and associated with significant mortality [20,41]. Among the common cardiac complications are acute myocardial infarction, new or worsening arrhythmia (most commonly atrial fibrillation) and new or worsening cardiac failure. While there are several risk factors for these CVEs, including severe pneumonia, older age, and pre-existing CV disorders, it is important to recognize that these events may also occur in relatively young, otherwise healthy individuals with no prior history of cardiac disease [20]. As mentioned above, the first description of cardiac complications in pneumococcal CAP was reported in 2007 [10], while in 2008, a case study was published of a 65-year-old woman with Takotsubo cardiomyopathy associated with pneumococcal sepsis [42]. More recently, a 15-year population-based study of patients with IPD (mean age of 54.2 years), many of whom had a significant co-morbidity, documented frequencies of myocardial infarction, pulmonary embolism, and new onset stroke in 1.5%, 1.3% and 1.1% of patients respectively [43]. Of those that had an echocardiogram during their infection, 35% were noted to have impaired ventricular function. Importantly, recent respiratory virus infection, especially influenza virus infection, is also a well-recognized risk factor for development of acute myocardial infection [44]. In this context, preceding influenza virus infection also represents a major risk for development of IPD [45], indicating that co-infection with these viral and bacterial pathogens augments the risk for development of acute CVEs. 

The importance of these cardiac complications occurring in patients with CAP is underscored by the fact that they are associated not only with a 5-fold increase in CAP-associated 30-day mortality [13], but also with increased mortality and heightened risk of CVEs even on long-term follow-up following hospital discharge [46]. One recent study evaluated the risk of heart failure in 4988 adults with CAP (both in- and out-patients) and no prior history of heart failure who were prospectively recruited and matched by age, sex and setting of treatment with up to 5 adult cases without pneumonia or heart failure who acted as controls [14]. Over a median of 9.9 years, a total of 11.9% of patients with a prior history of CAP developed incident heart failure compared with only 7.4% of controls (adjusted HR 1.61; 95% CI 1.44–1.81). The risk of heart failure in patients who had previously developed CAP was higher in patients >65 years compared to younger patients; nonetheless even in the younger cases the risk was raised and was greater than in the control group of patients. These authors highlighted not only the need to screen for, and to institute preventative strategies to modify risk factors for cardiac events in patients who have had CAP, but also to consider vaccination against pathogens such as the pneumococcus and influenza to prevent CAP [14,43].

In the case of pneumococcal CAP, compelling evidence, much of it experimental, has implicated a key role for pneumolysin (PLY) in the pathogenesis of myocardial damage and resultant CVEs associated with IPD. The mechanisms of PLY-mediated cardiotoxicity, as well as toxin-targeted neutralization strategies, represent the major thrusts of the remaining sections of this review. These are preceded by a brief consideration of toxin structure and reactivity. 

## 4. Structure and Biological Activities of Pneumolysin

The 53 kDa molecular weight, highly-conserved, major protein virulence factor of the pneumococcus, PLY, is composed of 471 amino acids arranged into four functionally discrete domains [1,47]. Domain 4**,** which contains the undecapeptide sequence that is largely conserved among all members of the bacterial cholesterol-dependent cytolysin (CDC) family, mediates binding of PLY to the eukaryotic cell membrane, while domains 1–3 collectively confer structural stability [1]. The toxin localizes to the cytoplasm of the pneumococcus where it is released either by autolytic mechanisms or by the action of bacteriolytic antibiotics such as β-lactam agents [1,47]. Unlike other bacterial CDCs, PLY is not actively secreted by the pneumococcus due to the absence of a typical N-terminal signal secretion leader sequence [1]. The toxin in its active form has, however, been detected in the cell wall of some serotypes of the pneumococcus, where it is likely to favor bacterial invasion and persistence [48,49]. In this context, export of toxin from the cytoplasm to the cell wall is dependent on the activity of the transmembrane accessory secretion (Sec) system, SecY2A2 [49]. 

The mechanisms involved in PLY-mediated pore formation have recently been described elsewhere [18,50]. Briefly, binding of CDCs to target cells involves generic mechanisms, which are critically dependent on the interaction of the common hydrophobic attachment region of domain 4 of the toxins with cholesterol head groups of the eukaryotic cell membrane [1]. This type of interaction of the toxins with membrane cholesterol involves three hydrophobic loops (L1–L3) in domain 4 [51] and appears to be achieved via the binding to cholesterol of a threonine-leucine pair located in the L1 loop [52]. These events precede insertion of the undecapeptide sequence into the membrane [51] and are a pre-requisite for subsequent oligomerization of toxin monomers, which is dependent on domains 1–3. In the case of PLY, this leads, in turn, to self-assembly of intermediary pre-pores followed by formation of membrane-spanning β-barrel pores of maximum diameter 400 Å consisting of 42 toxin monomers [53]. 

At high concentrations, PLY induces irreversible pore formation resulting in cytolysis of a variety of cell types, which vary somewhat with respect to sensitivity to the cytolytic actions of the toxin. These include erythrocytes, platelets, cells of both the innate and adaptive immune systems, cardiomyocytes, and various types of structural cells, especially epithelial cells, and endothelial cells, as described below. The major consequences of these cytotoxic activities of PLY are immunosuppression and tissue damage, both of which favor extra-pulmonary dissemination and persistence of the pneumococcus. At lower, sub-lethal concentrations, on the other hand, pore formation induced by PLY is transient and repairable, which, in the case of cells of the innate immune system and platelets, causes hyper-activation of the pro-inflammatory and pro-thrombotic activities of these cells. The most prominent mechanism underpinning these events appears to involve the influx of extracellular Ca^2+^ into cells exposed to sub-lytic concentrations of PLY, resulting in intracellular Ca^2+^ overload, efflux of K^+^, membrane depolarization and cellular hyper-reactivity [54,55,56]. Important examples of these events with respect to pro-inflammatory/pro-thrombotic mechanisms involved in the pathogenesis of organ dysfunction during severe pneumococcal infection include the following:

- potentiation of the generation of reactive oxygen species (ROS) and release of elastase by chemoattractant-activated human neutrophils, as well as production of prostaglandin E_2,_ leukotriene B_4_ and interleukin (IL)-8, and release of matrix metalloproteinase-9 by both unstimulated and chemoattractant-activated cells [54,57,58,59].
- activation of the Nod-like receptor protein 3 (NLRP3) inflammasome in murine dendritic cells, as well as human and murine neutrophils, resulting in cleavage of pro-IL-1β to the active form of this pro-inflammatory cytokine [55,60,61]
- induction of the formation of neutrophil extracellular traps (NETs) via a process of vital NETosis [62]. NET formation involves the extrusion of decondensed nucleosomes and granule-derived proteins into the extracellular milieu by neutrophils, a process which contributes to the intravascular host innate immune response via entrapment of blood-borne pathogens. However, if excessive and poorly controlled, the process of NETosis poses the potential hazard of generation of coronary thrombi by mechanisms described below
- activation of human platelets detected according to up-regulation of the expression of the adhesion molecule, CD62P (P-selectin), which is stored in platelet α-granules [56]
- activation of the formation of pro-inflammatory/prothrombotic neutrophil:platelet heterotypic aggregates, an event which is critically dependent on up-regulated expression of CD62P on platelets and interaction with its counter-ligand, P-selectin glycoprotein ligand 1 (PSGL-1), on neutrophils [63].

Although all of the aforementioned activities of PLY were derived from studies based on in vitro experiments, it is noteworthy that the final concentrations of recombinant PLY used in almost all of these (200 ng/mL and considerably lower), with one exception in which a fixed concentration of 500 ng/mL was used [55], were within the range of those detected in the cerebrospinal fluid of patients with proven pneumococcal meningitis, which achieved maximum values of around 700 ng/mL [64]. 

## 5. Direct Cardiotoxic Activity of Pneumolysin

Brown et al. in 2014 were the first to describe the seemingly key involvement of PLY in the pathogenesis of myocardial injury in mice experimentally infected with either the TIGR4 (serotype 4) or D39 (serotype 2) strains of the pneumococcus [15]. Experimental infection with the pneumococcus by either the intraperitoneal or intratracheal routes resulted in invasive disease associated with a linear increase in the numbers of bacteria in the blood of mice during the 12–30 h period post-infection. Bacteremia was strongly correlated with the development of cardiac damage and dysfunction according to detection of electrocardiogram aberrations and steadily increasing concentrations of circulating cardiac troponin-I. Histological analysis of dissected organs revealed the presence of randomly distributed microscopic lesions containing aggregates of pneumococci throughout the ventricular myocardium and a paucity of inflammatory cells, which was most evident with infection caused by the TIGR4 strain of the pneumococcus [15]. These microlesions were not, however, detected in liver, spleen, or kidney, seemingly consistent with a predilection of the pneumococcus for cardiac tissue [15]. In a small series of additional experiments (*n* = 2), which did not include electrophysiological assessments or measurement of cardiac troponin-I, injection of a bolus of recombinant PLY through the retro-orbital venous sinus, although causing “considerable signs of damage and inflammation within the cardiac microvasculature”, failed to cause myocardial microlesion formation at 24 h post-administration [15].

Mechanistic studies based on inactivation of the pneumococcal CbpA gene or the murine phosphorylcholine-binding PAF receptor gene, revealed the involvement of both pneumococcal adhesins in myocardial invasion, a finding which was strengthened by the observed protective effects of prior immunization of mice with recombinant CbpA [15]. Evidence in support of the key involvement of PLY in mediating myocardial damage, apparently by immune-quiescent, pro-apoptotic mechanisms, included the following: (i) protection against cardiac microlesion formation by either deletion of the PLY gene of the pathogen, or by prior immunization of mice with a PLY vaccine attenuated with respect to pore-forming activity (pneumolysoid); and (ii) the observed susceptibility of a cardiomyocyte cell line to the cytotoxic action of PLY in vitro. In addition, implementation of antimicrobial chemotherapy with a bacteriolytic agent (ampicillin) was associated with intense myocardial infiltration by immune/inflammatory cells, resulting in collagen deposition in resolving lesions and resultant scarring, which, as suggested by the authors, may underpin the development of delayed-onset CVEs in humans [15].

Importantly, histological analysis of cardiac sections from 3 simian immunodeficiency virus-infected male rhesus macaques, which had succumbed following experimental infection with a serotype 19F strain of the pneumococcus, as well as from 2 of 9 adult humans with fatal IPD, also revealed the presence of cardiac lesions [15].

Follow-up studies undertaken by members of the same research team revealed the following additional insights into the pathogenesis of myocardial injury associated with experimental IPD:

- myocardial invasion by the pneumococcus in the murine model of IPD is associated with strain-specific, PLY-mediated necroptosis (programmed necrotic cell death) of resident and infiltrating cardiac macrophages, which may contribute to the observed paucity of immune and inflammatory cells in microlesions [65]. In this context, it is noteworthy, as reported by others, that bacterial pore-forming toxins, including PLY, induce necroptosis of alveolar macrophages in a murine model of experimental pneumonia [66], which appears to be strain-specific [67]
- although CbpA and phosphorylcholine appear to be determinants of myocardial invasion via interaction of these adhesins with vascular endothelium, invasion of cardiomyocytes by the pneumococcus in vitro results from an alternative mechanism involving clathrin-mediated endocytosis of the pathogen [68]. In this setting, intracellular pneumococci were found to reside in the cytoplasm and cytoplasmic vacuoles, with resultant killing of infected cardiomyocytes mediated by exposure to PLY acting in concert with pneumococcus-derived hydrogen peroxide [68]
- infection of cardiomyocytes and/or macrophages or fibroblasts by the pneumococcus during experimental IPD results in intracellular proliferation of the pneumococcus and transition of the pathogen from a planktonic to a biofilm-forming phenotype, which is associated with formation of cardiac microlesions [69]. Acquisition of the biofilm-forming phenotype, in turn, is associated with an altered transcriptional profile of the pneumococcus, which results in high-level production of PLY and resultant elimination of cardiac macrophages [69]
- extrapolation of these findings to a scenario more representative of severe pneumococcal disease in humans was achieved by using a non-human primate (adult baboon, species *Papio cynocephalus*) model of IPD in which the TIGR4 strain of the pneumococcus is inoculated directly into the right middle lobe of the lung [70]. Using this model, pneumococcal bacteremia was found to result in myocardial invasion by the pathogen, cardiomyocyte death by both apoptotic and necroptotic mechanisms, cardiac dysfunction, and cardiac scarring in convalescent, antibiotic-treated animals [71].

Following the original report by Brown et al. in 2014 [15], Alhamdi et al. in 2015, using a model of intravenous experimental infection caused by various matched strains of the pneumococcus with normal and defective expression of PLY, confirmed the key involvement of the toxin in causing myocardial damage and mortality [16]. Myocardial damage was detected according to markedly elevated concentrations of circulating cardiac troponins-I and -T and was attenuated by pre-treatment of the animals with cholesterol-containing, PLY-sequestering liposomes and exacerbated by treatment with ampicillin [16]. Unlike Brown et al. [15], however, Alhamdi et al. [16] failed to detect any of the following: (i) involvement of CbpA in the pathogenesis of myocardial injury during experimental IPD; (ii) invasion of the myocardium by the pathogen; and (iii) histologic evidence of gross myocardial abnormalities. Another notable difference between the two studies was the observation by Alhamdi et al. that myocardial injury was mimicked by intravenous administration of biologically-active, recombinant, PLY, which, was associated with intramyocardial accumulation of inflammatory cells [16]. In addition, PLY at high concentrations was found to cause lysis, as opposed to apoptosis, of cardiomyocytes, while at lower sub-lytic concentrations, the toxin also caused significant contractile dysfunction of these cells in vitro [16]. This latter mechanism was associated with reversible, sub-lytic cardiomyocyte membrane pore formation, influx of extracellular Ca^2+^ and intracellular Ca^2+^ overload, with resultant efflux of K^+^ and membrane depolarization, events which, if operative in vivo, are likely to contribute to myocardial contractile dysfunction [16].

To reconcile the inconsistencies between the studies reported by Brown et al. [15] and Alhamdi et al. [16], Gilley et al. [65] proposed the following explanations: (i) differences in the routes of experimental infection (intraperitoneal/intratracheal vs. intravenous); (ii) strain-specific variations in virulence and pathogenesis (predominantly TIGR4 vs. D39); and (iii) the greater sensitivity of the indirect immunofluorescence procedure used by Brown et al. for histological detection of pneumococci [15]. Irrespective of differences in the proposed mechanisms of pathogenesis of IPD-related, secondary cardiac dysfunction, both research groups have, however, clearly and impressively documented the key role of PLY in the pathogenesis of experimental, IPD-associated acute myocardial injury. This, in turn, has triggered renewed interest in the development of PLY-targeted therapeutic and preventive strategies.

## 6. Indirect Pro-Inflammatory/Pro-Thrombotic Potential of Pneumolysin in the Pathogenesis of Cardiac Damage and Dysfunction

Although largely unexplored, it is probable that the pro-inflammatory/pro-thrombotic interactions of PLY with platelets and neutrophils in particular are also involved in the pathogenesis of myocardial damage during severe pneumococcal infection. In this context, it is noteworthy that platelets are increasingly recognized as cells which bridge innate and adaptive immune responses [72,73], while platelet-driven, hyper-activation of neutrophils in particular has been implicated in the pathogenesis of various types of thrombotic disorder [73,74].

With respect to the involvement of PLY in mediating activation of platelets and neutrophils, exposure of both cell types to the toxin is associated with PLY-mediated sub-lytic pore formation and influx of Ca^2+^. In the case of platelets, these events result in up-regulation of expression of the pro-adhesive integrin GPIIb/IIIa, as well as CD62P, which induce homotypic platelet aggregation and neutrophil:platelet aggregation respectively [56,63,75]. In the case of neutrophils, exposure of these cells to the purified toxin has been reported to promote induction of vital NETosis [62], which is potentiated by neutrophil-adherent platelets via the presentation of pro-NETotic high mobility group box 1 protein (HGMB1) to neutrophils [76]. NETs, in turn, promote thrombosis by several mechanisms, including expression of tissue factor [77], inactivation of endogenous anticoagulants [78], binding and activation of platelets [79], and activation of the intrinsic pathway of coagulation [80]. The consequence of these NET-driven, pro-thrombotic events is activation of intravascular coagulation, both venous and arterial, increasing the risks of release of emboli and direct arterial occlusion with resultant ischemia and possibly infarction of myocardial tissues [74,81]. In this context, it is noteworthy that severe influenza A virus infection is also associated with a high level of intravascular NETosis [82], suggestive of a possible augmentative interaction with PLY in the pathogenesis of acute CVEs.

Although the specific involvement of PLY in causing platelet activation and NETosis during IPD remains unexplored, it is, however, noteworthy that significant platelet activation and hyper-reactivity have been demonstrated in a porcine model of experimental IPD [83], while acute CVEs in patients with severe CAP have been reported to be associated with an activated platelet phenotype [84].

**Figure 1:**
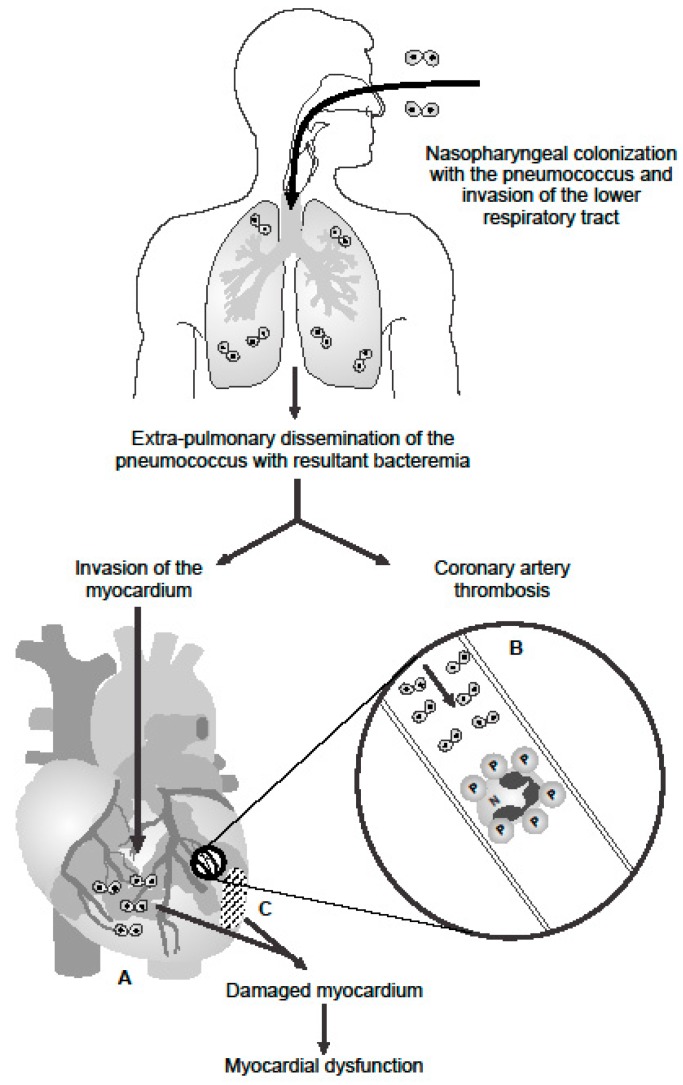
Proposed mechanisms involved in the pathogenesis of pneumolysin-mediated myocardial injury during invasive pneumococcal disease in humans. Following nasopharyngeal colonization, invasion of the lower respiratory tract, extra-pulmonary dissemination, and cardiac invasion by the pneumococcus (⨀⨀–symbol represents diplococci), intra-myocardial and intravascular release of pneumolysin (PLY) by the pathogen results in A: PLY-mediated death and dysfunction of cardiomyocytes; B: intravascular activation of platelets and neutrophils with resultant formation of pro-thrombotic/pro-NETotic neutrophil (N):platelet (P) aggregates (as illustrated in the magnification of an affected coronary arteriole/artery); and C: development of myocardial damage and dysfunction.

The aforementioned direct cytotoxic and indirect pro-inflammatory/pro-thrombotic mechanisms by which PLY may promote IPD-associated myocardial damage and dysfunction in humans are summarized in Figure 1. It must be noted, however, that evidence favoring the existence of these mechanisms is largely based on experimental animal models of IPD supported by in vitro laboratory studies. We also concede that other virulence mechanisms may be involved in the pathogenesis of IPD-related CVEs. These may be unraveled following identification of possible associations of strains of the pneumococcus which produce non-hemolytic variants of PLY with development of CVEs.

## 7. Update on Pneumolysin-Targeted Therapeutic Strategies

Several notable developments have occurred since the publication of our recent review on this topic [18], specifically in relation to macrolide antibiotics, and the identification of novel low molecular weight antagonists of PLY, as well as the early clinical evaluation of CDC-sequestering liposomes and fully-humanized, toxin-neutralizing monoclonal antibodies in patients with severe pneumonia.

### 7.1. Macrolide Antibiotics

Macrolide and macrolide-like agents counter the harmful actions of PLY, as well as those of several other bacterial pore-forming toxins, by a dual mechanism of action involving primary antimicrobial activity and secondary, predominantly neutrophil-targeted, anti-inflammatory activity [18]. This unusual combination of therapeutic activities appears to distinguish macrolides from other classes of antibiotic inhibitors of bacterial protein synthesis.

#### 7.1.1. Macrolides and Inhibition of the Synthesis of Pneumolysin

With respect to antimicrobial activity, macrolides, which are essentially bacteriostatic, target the bacterial 50S ribosome, binding to the peptide exit tunnel and interfering with peptide chain elongation [85], a mechanism which underpins their efficacy as inhibitors of the synthesis of PLY [86]. In the case of the antimicrobial chemotherapy of patients hospitalized for severe CAP, many [87], but not all [88], national guidelines recommend administration of the combination of a bactericidal β-lactam agent and a macrolide. Although remaining to be conclusively established in the clinical setting, support for this approach is based on the contention that the macrolide, via attenuation of synthesis of PLY and other bacterial protein toxins, alleviates potentially harmful excessive inflammatory responses secondary to administration of the bacteriolytic β-lactam agent [18,87].

In his recent editorial on the topic, Waterer, while conceding that consensus has not been reached, strongly advocates the benefits of combination therapy of patients with severe CAP [87] and cites two well-controlled clinical studies in support of this position. Firstly, a study comparing the impact on mortality of differences in strategies related to both timing of administration and type of antibiotic therapy in patients who were admitted to hospital ICUs with proven severe pneumococcal pneumonia during the periods 2001–2002 and 2008–2013 [89]. The authors reported that early administration of antibiotics and the use of combination therapy, most commonly the addition of a macrolide (particularly azithromycin) to a cephalosporin, increased over time and was clearly associated with improved survival [89]. The second and more recent study reported by Okumura et al. reported on differences in 30-day mortality rates between patients with all-cause CAP treated with either β-lactam monotherapy or β-lactam/macrolide combination therapy [90]. The pneumococcus was the most commonly identified pathogen in both groups of patients, while the mortality rates were 13.8% and 1.8% (*p* < 0.001) in those receiving monotherapy and combination therapy respectively [90].

In an earlier review, we raised the issue of the possible importance of both the timing and sequence of administration of the β-lactam and macrolide antibiotics in patients with severe CAP, contending that administration of the macrolide prior to the β-lactam may be beneficial, particularly in the context of pneumococcal CAP [91]. This issue has recently been addressed by Peyrani et al. who reported that administration of the macrolide before, as opposed to after, the β-lactam antibiotic was associated with significant decreases in time to clinical stability and length of hospital stay (*p* = 0.011 and *p* = 0.002 respectively), but not mortality [92]. These findings led the authors to speculate that “the beneficial effect of macrolides in hospitalized patients with CAP may occur only if administered before beta-lactams” [92]. In a similar type of study, Metersky et al. reported that the combined in-hospital mortality/hospice discharge rates of patients with severe CAP treated with an intravenous macrolide either at least one hour before or after a β-lactam were 6.3% and 9.3% respectively [93]. Although not attaining statistical significance, the authors concluded that given the large confidence intervals, these differences do “allow for the possibility of a clinically significant benefit” [93] and that further studies are warranted.

In his editorial on this issue, Waterer describes the data in favor of the administration of combination β-lactam/macrolide therapy to patients with severe CAP as being “overwhelming” and advocates administration of “a macrolide followed a short time later by a third-generation cephalosporin please, preferably getting both within 2 h of presenting” [87].

#### 7.1.2. Anti-inflammatory Activities of Macrolides

The secondary anti-inflammatory activities of macrolides, which are unrelated to antimicrobial activity, have been considered in a recent review focused specifically on possible antagonism of the pro-inflammatory activity of PLY [18] and are covered only briefly here. In this context, the neutrophil appears to represent the primary cellular target of macrolides, which act by suppressing the activities of various cytosolic transcription factors involved in activation of the synthesis of the neutrophil-activating chemokine, IL-8, as well, as the pro-inflammatory cytokines, IL-17A and TNF-α, by both immune/inflammatory and structural cells [reviewed in 18]. More recently, the 15-membered macrolide antibiotic, azithromycin, as well as chloramphenicol and gentamicin [94,95], but not the β-lactam agents amoxicillin [95] and cefotaxime [95], or the macrolide-like agent, clindamycin [96], have been reported to inhibit NET formation in vitro. In the case of azithromycin, which is commonly used in the combination therapy of CAP, these inhibitory effects on NETosis were detected at concentrations of 0.5 µg/mL, achieving statistical significance at 10 µg/mL (the next higher concentration tested) [94]. This latter concentration is attainable in blood following intravenous administration of a 4 g dose of this agent [97]. The suppressive effects of azithromycin on NETosis were associated with, and were possibly related to, decreased neutrophil membrane-associated oxidative metabolism [94]. These findings, however, differ from those of Konstantinidis et al., who reported that both azithromycin and the 14-membered macrolide agent, clarithromycin, at therapeutically-relevant concentrations, induce statistically significant, spontaneous NETosis in vitro, as well as ex vivo, following antimicrobial chemotherapy with these agents [98]. The authors speculate that macrolide-mediated induction of NETosis may contribute to host defense against microbial pathogens [98]. Clearly, additional, definitive studies are necessary to clarify the potentially important issue of possible macrolide-mediated modulation of NETosis, particularly that induced by PLY.

To date, however, no studies have addressed the very important issue of the role of combination therapy, specifically inclusion of a macrolide, in attenuating the incidence of acute, as well as delayed-onset, CVEs in patients with severe IPD.

### 7.2. Low Molecular Weight Antagonists of the Pore-Forming Action of Pneumolysin

#### 7.2.1. Naturally Occurring Antagonists

The recent literature on this topic has largely originated from a research program undertaken by a Chinese research group. In this context, their research has focused on the identification and isolation of antagonists of PLY isolated from traditional Chinese medicinal herbs. These include various phytosterols, polyphenols, bioflavonoids, and naphthoquinones. The various PLY antagonists identified, none of which was found to possess antimicrobial activity, effectively attenuated the hemolytic activity of PLY, as well as the injurious effects of the toxin on an alveolar epithelial cell line in vitro [99,100,101,102,103,104,105,106,107]. In addition, subcutaneous administration of these various natural antagonists of PLY resulted in significantly improved survival in a murine model of intranasal pneumococcal lung infection [99,100,101,102,103,104,105,106,107]. The various members of each category of natural antagonists of PLY are summarized as follows:

- ***phytosterols*:** β-sitosterol was initially identified as a cholesterol-mimic, interacting with the conserved cholesterol-binding site located on domain 4 of PLY, with potency comparable with that of cholesterol [99]. A follow-up, mechanistic study, using a molecular dynamics simulation approach, revealed two additional phytosterols which also antagonized the membrane-binding activity of PLY *viz.* campesterol and brassicasterol, with the former demonstrating activity comparable with that of β-sitosterol and the latter being somewhat less potent [100]
- ***polyphenols*:** verbascoside (a caffeoyl polyethanol glycoside) and the catechin, epigallocatechin gallate, are naturally-occurring polyphenol anti-oxidant antagonists of PLY, which have demonstrated almost complete attenuation of the hemolytic activity of the toxin at concentrations of about 2.0 and 2.7 µg/mL respectively [101,102]. Unlike the phytosterols, the polyphenol antagonists appear to interfere with the oligomerization of PLY monomers on the target cell membrane via binding to the cleft between domains 3 and 4 of the toxin molecule [101,102]
- ***bioflavonoids*:** three bioflavonoids *viz.* apigenin (4′,5,7-trihyddroxyflavone) [103], morin [2-(2,4-dihydroxyphenol)-3,5,7-trihydroxychromen-4-one] [104] and amentoflavone {8-[5-(5,7-dihydroxy-4-oxo-chromen-2-yl)-2-hydroxy-phenyl]-5,7-dihydroxy-2-(4-hydroxyphenyl)chromen-4-one} [105] have also been found to attenuate the harmful activities of PLY. All three agents were found to cause inhibition of the hemolytic activity of PLY at concentrations of around 16 µg/mL by apparent interference with the oligomerization of toxin monomers [103,104,105]
- ***naphthoquinones:*** two members of this class of chemical agent *viz.* shikonin {also known as alkanin: 5,8-dihydroxy-2-[(1*S*)-1-hydroxy-4-methylpent-3-en-1 yl]naphthalene-1,4-dione} [106] and juglone (5-hydroxy-1,4-naphthalenedione) [107] have also been reported to inhibit the pore-forming activity of PLY via interference with toxin oligomerization. Shikonin demonstrated superior activity, causing almost complete inhibition of the hemolytic activity of PLY at concentrations ≥1 µg/mL [106]. Although not addressed in these studies [106,107], it is noteworthy that 1,4-naphthoquinones possess pro-oxidative properties that may promote oxidative inactivation of membrane pore formation mediated by PLY and other CDCs via oxidative inactivation of the essential cysteine residue located in the undecapeptide membrane insertion region of the toxin [51].

To our knowledge, none of these naturally-occurring antagonists of PLY, most of which, as mentioned above, have demonstrated protective efficacy in murine models of experimental pneumococcal lung injury, has entered the early stages of evaluation in the clinical setting of severe pneumococcal disease.

#### 7.2.2. Magnesium Chloride

Hupp et al. recently described the beneficial effects of magnesium chloride (MgCl_2_), administered intraperitoneally at a therapeutically-relevant dose of 500 mg/kg body weight, in protecting young rats against the neurotoxic/neuroinflammatory effects of intracerebral injection of recombinant PLY, as well as mice, following experimental infection with strain D39 of the pneumococcus [50]. In the shorter duration experiments with PLY, MgCl_2_ was administered as a single dose either before, or after the toxin, while in the murine experimental pneumococcal meningitis model the divalent cation was administered as three doses at 12-hourly intervals, the first at 30 min before induction of infection [50]. In the PLY experimental system, administration of MgCl_2_ was accompanied by significant attenuation of cerebral edema, and with decreased synaptic loss and increased survival in the murine model of experimental meningitis [50]. Mechanistic studies with the purified toxin and isolated glial cells in vitro demonstrated that the protective effects of MgCl_2_ were not attributable to interference with the membrane-binding activity of the toxin, or with the conductance of PLY pores, but were seemingly related to delayed pore formation [50]. Possible alternative or interactive mechanisms include competitive interference with PLY-mediated Ca^2+^ influx and/or uptake of Ca^2+^ via endogenous Ca^2+^ channels [50]. 

Given the safety of Mg^2+^ and its current therapeutic application in several clinical settings of non-infective origin, the authors propose that the findings of their “meningitis model identifies magnesium as a promising candidate for adjunctive treatment of pneumococcal meningitis together with antibiotic therapy” [50]. However, the potential of Mg^2+^ to protect against myocardial injury in the settings of experimental and clinical IPD remains to be established.

#### 7.2.3. Liposome-Based Strategies

As mentioned above, Alhamdi et al. reported that liposomes containing a 1:1 mixture of cholesterol:sphingomyelin (66 mol % cholesterol), initially described by Henry et al. as a strategy to counter the harmful effects of bacterial pore-forming toxins [108], significantly attenuated myocardial injury when administered intravenously to mice at a dose of 100 mg/kg body weight 30 min prior to initiation of experimental IPD [16]. Importantly, cholesterol-containing liposomes of this type are currently undergoing early clinical evaluation, the prototype agent being the cholesterol-containing liposomal agent, CAL02, manufactured by the Swiss biotechnology company, Combioxin SA [109]. CAL02 is described as “a first-in-class non-antibiotic liposomal drug that neutralizes a large panel of bacterial toxins, protects against infection severity and complications, and improves antibiotic efficacy” [109]. Having successfully completed initial safety evaluation in patients with severe pneumococcal pneumonia, this agent, administered intravenously at a dose of 16 mg/kg body weight, is currently undergoing phase IIb evaluation (safety, tolerability, pharmacodynamics, efficacy) in a randomized double-blind, placebo-controlled, multicenter clinical trial in a much broader population of patients with severe infections [110]. 

Of the various strategies described in this section of the review, CAL02-based studies appear most likely to deliver the earliest evidence in respect of the clinical efficacy (or lack thereof) of PLY-targeted therapeutic approaches, including prevention of IPD-associated CVEs. 

#### 7.2.4. Monoclonal Antibody-Based Strategies

An earlier study undertaken by García-Suárez et al. demonstrated that the intravenous administration of two murine PLY-neutralizing monoclonal antibodies (MAbs) significantly increased the mean survival time of mice infected intranasally with a lethal dose of the pneumococcus [111]. It is only fairly recently, however, that the field of passive immunotherapy of infections caused by toxin-producing bacterial pathogens has gained momentum coincident with the development of fully humanized monoclonal antibody technology [112]. In this context, diseases caused by extracellular toxin-producing bacterial pathogens, most prominently *Staphylococcus aureus*, as well as *Bacillus anthracis*, *Clostridium difficile*, *Escherichia coli* and *Pseudomonas aeruginosa*, represent the major targets of MAb-based immunotherapy, with at least nine of these therapeutic MAb approaches currently undergoing phase I/II clinical evaluation [112].

With respect to therapeutic targeting of bacterial pore-forming toxins, the most significant recent advances have focused on antibiotic-resistant, toxin-producing strains of *S. aureus*, which cause severe necrotizing pneumonia associated with considerable mortality. In this context, the development of the therapeutic MAb preparation, ASN100, by the Austrian biotechnology company, Arsanis Biosciences GmbH, is particularly noteworthy [112,113]. ASN100 comprises two fully humanized monoclonal antibodies, ASN-1 and ASN-2, which collectively target six different staphylococcal pore-forming cytolysins *viz.* α-hemolysin, γ-hemolysins AB, and CB, and the leukocidins, LukED, LukGH and Panton-Valentine leukocidin [112,113,114,115,116]. This therapeutic MAb combination is currently undergoing evaluation in a phase II clinical trial focused on the prevention of ventilator-associated pneumonia in high-risk patients admitted to ICUs [112,113].

Given the magnitude of the threat to public health posed by IPD, particularly in the elderly, together with the potential of PLY as a broad spectrum therapeutic target, the seemingly slow pace of MAb-based, PLY-targeted immunotherapy as an adjunct to antibiotics is surprising. In this context, it is, however, noteworthy that an Arsanis anti-pneumococcal MAb, ASN400, is described as being in the “discovery” phase of development, although its therapeutic target does not appear to have been revealed [113].

#### 7.2.5. Indirect Strategies to Counter the Harmful Activities of Pneumolysin

The therapeutic potential of anti-inflammatory and anti-thrombotic adjunctive strategies based on administration of corticosteroids, statins or anti-platelet therapies, which may also counter the harmful, pro-inflammatory/pro-thrombotic activities of PLY in patients with severe CAP have been reviewed elsewhere [18,19,20,21] and are not covered here. However, more recent developments with respect to the identification of novel, indirect, toxin-targeted, potential adjunctive therapeutic strategies which merit mention include the following: 

***Vasculotide***: This agent, developed for the treatment of ALI/ARDS and acute kidney injury, is a synthetic, polyethylene glycol-clustered peptide mimetic of angiopoietin-1, which alleviates endothelial dysfunction via activation of Tie receptors [117]. The therapeutic potential of vasculotide in preventing PLY-mediated lung injury was recently described by Gutbier et al. [118]. These authors reported that vasculotide pre-treatment of murine lung endothelial cell monolayers and isolated lungs protected against PLY-mediated disruption of barrier function and increased vascular permeability respectively [118]. In addition, using a murine model of transnasal pneumococcal infection, intravenous administration of vasculotide at doses of 100, 200 and 500 nanograms at 22, 34 and 46 h post-infection resulted in statistically significant, dose-related reductions in lung permeability without affecting pulmonary inflammatory responses and bacterial burden [118]. The authors concluded that vasculotide “may provide a novel therapeutic perspective for reduction of permeability in pneumococcal pneumonia-induced lung injury” [118].

***Tumor-necrosis factor (TNF)-α-derived TIP peptide***: As with vasculotide, the major potential clinical application of TNF-derived TIP circular peptide, which is based on the lectin-like domain of TNF-α, is in reducing pulmonary edema in patients with ALI/ARDS [119]. TNF-derived TIP peptide-mediated clearance of alveolar fluid is achieved via activation of the type II alveolar epithelial cell amiloride-sensitive sodium channel (ENaC) [119]. In this context, a recent report [120] by a research group, which had previously recognized the potential of TIF-derived TIP peptide to ameliorate PLY-mediated lung injury [17], is noteworthy. These authors reported that isolated human lung microvascular endothelial cells, like epithelial cells, express all three subunits of the ENaC [120]. They also observed that deletion of the α-subunit of the endothelial ENaC significantly increased sensitivity to PLY-mediated hyper-permeability and barrier dysfunction, while TNF-derived TIP peptide treatment of endothelial cells with an intact ENaC protected against the damaging actions of PLY [120]. The authors concluded that selective targeting of ENaC, in addition to sustaining alveolar epithelial cell function, represents a novel strategy to improving the barrier function of the capillary endothelium in severe pneumonia [120]. This contention is supported by the findings of a recently completed clinical trial designed to assess the efficacy of an orally inhaled TNF-derived TIP peptide, AP301, on alveolar fluid clearance in intubated, mechanically-ventilated ICU patients, mostly with severe pneumonia [121]. The findings of this trial indicated reduction of the extravascular lung water index in patients with high (≥11) sequential organ failure assessment (SOFA) clinical scores [122]. 

The various PLY-targeted strategies covered in this section are summarized in Table 1. Most of these are also likely to be effective against other types of bacterial CDCs, while agents such as vasculotide, TNF-derived TIP peptide and especially macrolide antibiotics, are likely to be broadly operative against bacterial pore-forming toxins. Of those which specifically target CDCs, CAL02, as mentioned above, is the most advanced with respect to clinical evaluation of therapeutic targeting of PLY.

## 8. Pneumolysin as a Candidate Vaccine Antigen

Currently available pneumococcal vaccines, of which there are two types, target the serotype-specific, anti-phagocytic capsule of the most common disease-causing 97 serotypes of the pneumococcus [123]. The older pneumococcal polysaccharide vaccine 23 (PPV23) consists, as the name implies, of purified polysaccharides from 23 prevalent serotypes of the pneumococcus and is registered for use in adults and children ≥2 years of age [123,124]. Pneumococcal conjugate vaccines (PCVs) represent the second, more-recently developed type of pneumococcal vaccine, which, unlike the T helper lymphocyte-independent PPV23 vaccine, promote T cell-enhanced, anti-capsular antibody-mediated immune responses. Consequently, PCVs have improved immunogenicity relative to PPV23 and are registered for use in infants, children, and adults [123,124]. PCV13 is the current front-runner in this category of pneumococcal vaccines and is now administered routinely as a component of national childhood immunization programs globally [123,124].

Given the major burden of disease that the pneumococcus presents, it appears appropriate to consider pneumococcal vaccination for the prevention of severe infection and associated CVEs in those at highest risk. However, only PPV23, because of its extended availability, appears to have been evaluated in this context, albeit with uncertain efficacy [reviewed in 20]. This issue, specifically in the case of delayed-onset CVEs, may, however, be resolved on completion of an ongoing, randomized clinical trial [125].

Although the protective efficacy of PCV13 in particular is beyond doubt, the limited serotype coverage provided by PCVs, as well as PPV23, represents a well-recognized limitation of these vaccines, favoring nasopharyngeal colonization with non-vaccine serotypes of the pneumococcus [123]. Concerns surrounding restricted serotype coverage of polysaccharide-based pneumococcal vaccines have, in turn, provided impetus for the development of new generation, broadly cross-protective, recombinant protein vaccines based on highly conserved pneumococcal proteins [123,126]. In this context, PLY, given its broad expression across all pneumococcal serotypes, together with its role in colonization and disease pathogenesis, particularly its involvement in myocardial damage, is recognized as a prime candidate vaccine antigen [123,126]. PLY-based vaccines contain the toxin as a recombinant pneumolysoid, which is attenuated with respect to cytotoxicity via the introduction of point mutations into the *ply* gene. PLY-based vaccines may contain the pneumolysoid alone, or in combination with other highly-conserved recombinant pneumococcal proteins, or in combination with PCVs [123]. Pipeline pneumolysoid-containing vaccines which have reached the phase II/III stages of clinical evaluation include: (i) the Sanofi Pasteur common protein vaccine, which also contains two other recombinant pneumococcal proteins *viz.* polyhistidine triad protein D (PhtD) and pneumococcal choline-binding protein A (PcpA); and (ii) the GlaxoSmithKlline (GSK) Biologicals combined PCV10/pneumolysoid/PhtD vaccine [123,126]. 

Despite successful completion of early stage clinical evaluation, the protective efficacy of these novel pneumolysoid-containing vaccines, especially in the context of prevention of IPD and associated CVEs, remains to be established.

## 9. Conclusions

Although not yet proven in the clinical setting of IPD, the evidence implicating PLY in the pathogenesis of myocardial damage, albeit largely derived from experimental animal models of IPD, is compelling and may underpin the occurrence of both acute and delayed-onset CVEs in patients with severe CAP. Although other mechanisms may participate, it is the direct cytotoxic and immunosuppressive activities, as well as the indirect pro-inflammatory/pro-thrombotic activity of the toxin, which appear to be primarily involved in mediating cardiac damage and dysfunction. Notwithstanding the role of macrolides in inhibiting production of the toxin, several other PLY-targeted strategies which may attenuate the development of IPD-associated CVEs are currently in the advanced stages of clinical evaluation. These include cholesterol-containing liposomes and TNF-derived TIP peptide. Others such as low molecular weight, natural product antagonists and vasculotide are presently in the pre-clinical stages of evaluation. Somewhat disappointingly, however, the development of PL-targeted, therapeutic, fully humanized monoclonal antibodies is lagging behind that of other diseases caused by pore-forming toxin-producing bacterial pathogens. Although showing promise, the preventive potential of PLY-targeted vaccines remains to be established. Finally, recent insights into the mechanisms of PLY-mediated cardiac damage and dysfunction, together with initiatives aimed at development of novel PLY-targeted therapeutic and preventive strategies, are likely to impact significantly on the future management of patients with IPD.

## Figures and Tables

**Table 1:** Recognized and potential pneumolysin (PLY)-targeted therapeutic strategies.

Type	Mechanism	Status	Refs
Macrolide antibiotics	Inhibition of synthesis of PLY	Component of combination antibiotic therapy	[86]
Phytosterols (β-sitosterol; campesterol; brassicasterol)	Membrane binding of PLY	Pre-clinical	[99,100]
Polyphenols (verbascoside; epigallocatechin gallate)	Oligomerization of PLY monomers	Pre-clinical	[101,102]
Bioflavonoids (apigenin; morin; amentoflavone)	Oligomerization of PLY monomers	Pre-clinical	[103,104,105]
Naphthoquinones (shikonin; juglone)	Oligomerization of PLY monomers	Pre-clinical	[106,107]
Magnesium chloride	Delays pore formation; others	Pre-clinical with respect to severe pneumococcal disease	[50]
Liposome-based (CAL02)	Membrane binding of PLY	Phase IIb clinical evaluation	[16,108,109,110]
Monoclonal antibody-based	Membrane binding and inhibition of oligomerization depending on target epitope on PLY	Pre-clinical	[111,113]
Angiopoietin-1 mimetic (vasculotide)	Protects against PLY-mediated endothelial barrier dysfunction	Pre-clinical with respect to severe pneumococcal disease	[118]
TNF-derived TIP peptide	Activates the amiloride-sensitive sodium channel in epithelial and endothelial cells	Phase IIa trial recently completed	[119,120,121,122]
